# Molecular and biochemical analysis of the castor caruncle reveals a set of unique genes involved in oil accumulation in non-seed tissues

**DOI:** 10.1186/s13068-019-1496-6

**Published:** 2019-06-24

**Authors:** Xia Wan, Qing Liu, Bei Dong, Sapna Vibhakaran Pillai, Feng-Hong Huang, Surinder P. Singh, Xue-Rong Zhou

**Affiliations:** 1 Oil Crops Research Institute, Chinese Academy of Agricultural Sciences, Wuhan, 430062 People’s Republic of China; 2 CSIRO Agriculture & Food, PO Box 1700, Canberra, ACT 2601 Australia; 3 Key Laboratory of Biology and Genetic Improvement of Oil Crops, Ministry of Agriculture, Wuhan, 430062 People’s Republic of China; 4 Key Laboratory of Oilseeds Processing, Ministry of Agriculture, Wuhan, 430062 People’s Republic of China; 5 Hubei Key Laboratory of Lipid Chemistry and Nutrition, Wuhan, 430062 People’s Republic of China

**Keywords:** Castor bean, Caruncle, Non-seed oil accumulation, Transient expression, Transcriptome

## Abstract

**Background:**

With the increasing demand for vegetative oil and the approach of peak seed oil production, it is important to develop new oil production platforms from non-seed tissues. Castor bean (*Ricinus communis*) is one of the crops for vegetable oil for industrial applications with yield around 1.4 ton oil per hectare produced in seed. The castor caruncle is a non-seed tissue attached to seed.

**Results:**

Caruncle accumulates up to 40% oil by weight in the form of triacylglycerol (TAG), with a highly contrasting fatty acid composition when compared to the seed oil. Biochemical analysis indicated that the caruncle synthesizes TAGs independent of the seed. Such non-seed tissue has provided an excellent resource for understanding the mechanism of oil accumulation in tissues other than seeds. Transcriptome analysis revealed the key members of gene families involved in fatty acid synthesis and TAG assembly in the caruncle. A transient expression assay of these selected genes resulted in a 20-fold increased TAG accumulation in leaves.

**Conclusions:**

Castor caruncle utilizes an independent system to synthesize TAGs. Results provide the possibility of exploiting caruncle gene set to engineer oil production in non-seed tissues or microbes.

**Electronic supplementary material:**

The online version of this article (10.1186/s13068-019-1496-6) contains supplementary material, which is available to authorized users.

## Background

Vegetable oils are mainly extracted from seeds of soybean, rapeseed/canola, and sunflower, as well as from non-seed tissues, such as the mesocarp of oil palm, olive and avocado. The current world production of plant oil is about 190 million tons [1], which has doubled since 2000. However, the global demand for vegetable oils is rapidly increasing, and at a fast pace than agriculture can typically deliver, which puts significant pressure on food, feed and biofuel production system. Recent scientific efforts have been directed towards an alternative approach to make distinct type of vegetable oils in diverse non-seed tissues.

The accumulation of high levels of oils in high biomass vegetative tissues such as leaves rather than seeds has been considered as a promising strategy to meet the increasing demands for vegetable oils [2–4]. Vegetable oils are mainly comprised of TAGs which typically account for only less than 1% of total leaf lipids [5]. In general, plant leaves can synthesize fatty acid (FA), membrane lipids, diacylglycerol (DAG), and trace amounts of TAGs. Leaves normally do not accumulate high levels of storage lipids because their function is highly specialized for photosynthesis [6]. DAG is the precursor for both TAGs and membrane lipids. In leaves, the majority of DAG is converted into membrane lipids rather than TAGs [7]. Several biotechnology approaches to engineer oil production include increasing the flux of carbon into fatty acid and acyl-coenzyme A (CoA) synthesis, disruption of pathways competing with TAG synthesis, enhancing TAG assembly, decreasing TAG turnover, and stabilizing lipid droplets. These have been applied to boost the TAG content in leaves, with the highest TAG production being over 30% to the dry weight in engineered tobacco leaves [8, 9]. These studies provide strong evidence that engineering of vegetative biomass, especially in leaves, to produce oils is feasible.

In nature, there are diverse plants that accumulate oil in non-seed tissues. In addition to oil palm, olive and avocado, the mesocarp layer of Chinese tallow (*Triadica sebifera*) accumulates 80% oil by dry weight [10]. The outer surface layer of bayberry fruit (*Myrica rubra*) accumulates 32% wax by dry weight [11], mostly comprised of TAGs [12]. The stolon tuber of yellow nutsedge (*Cyperus esculentus*) contains 22–30% oil by dry weight [13, 14]. Oil firewood (*Tetraena mongolica*) stores about 4.6% TAGs by dry weight in stem tissues, mostly in the phloem cells, which contain about 9% TAGs [15]. Other specialized tissues like elaiosomes (‘oil body’ in Greek) are oil-rich structures attached to seeds in some plant species selected by nature for seed dispersal [16]. The elaiosome is a fleshy tissue that also plays a role in seed dehydration, rehydration and germination [17]. Although elaiosomes have been generally described as lipid-rich tissues, only a few studies have investigated the lipid content or fatty acid profile, while the majority has focused on seed dispersal by ants [18]. The elaiosome structure in castor bean is an oil-rich non-seed tissue called a caruncle. Castor bean is a tropical perennial shrub that originated in Africa but is now cultivated in many tropical and subtropical regions around the world. The seed oil content is around 60% of the dry weight, approximately 90% of which is composed of ricinoleic acid (RA, 12OH–C18:1^∆9^) [19]. RA and its derivatives are widely used for industrial products such as lubricants, plastics and surfactants.

The mechanism of RA production and oil accumulation in castor bean seed have been well documented [20–22]. By contrast, much less attention has been paid to caruncles, likely due to the mass of the caruncle being much smaller than the seed, and hence not making it an attractive resource for oil production. Detailed cellular structure of the various developmental stages ranging from pollination to maturation of the caruncle tissue which are closely related to that of the seed has been described by Bianchini and Pachini [17]. The caruncle consists of epidermal and parenchyma cells which accumulate storage lipids in the form of lipid droplets from 40 days after pollination onward and abundant lipid reserves were observed at maturity [17]. Therefore, caruncles might be suitable for studying the mechanism of oil accumulation in non-seed tissues, which may provide additional insights into how different enzymes involved in TAG assembly function in diverse tissues. In this study, we demonstrated that the TAG content of the caruncles reached up to 40% of the dry weight. In addition, fatty acids and TAGs were independently synthesized in caruncles. Therefore, we tried to characterize the functions of key genes and transcriptional factors specialized for fatty acid synthesis and TAG accumulation in caruncles. These highly expressed non-seed genes and transcriptional factors may be employed to increase non-seed oil accumulation.

## Results

### Oil accumulation and sugar content in developing castor seeds and caruncles

**Fig. 1:**
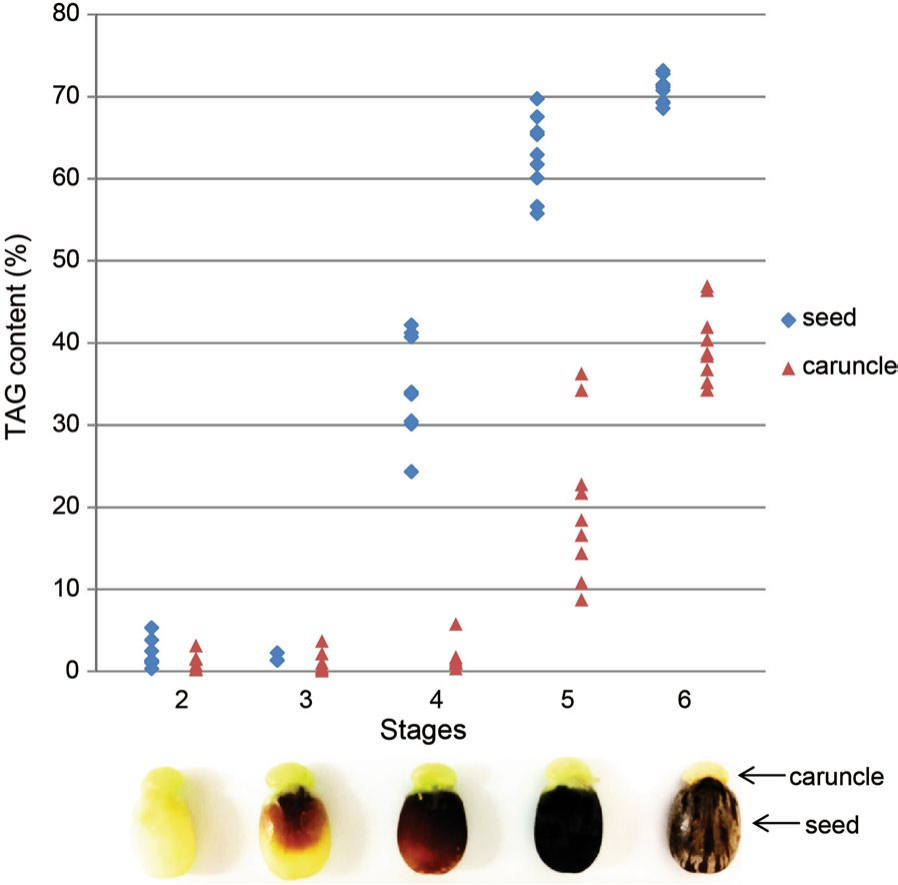
Oil content in castor seed and the caruncle during the seed development. Oil contents of individual seed and the attached caruncle were measured by nuclear magnetic resonance (NMR) as a percentage of dry weight. *n* ≥ 6

Castor seeds are rich in oil which is mainly stored in the endosperm. The caruncle is a unique tissue attached outside the seed. During seed development, which was divided into five stages based on morphologic characteristics [23] with the mature seed designated as stage 6, oil content of the seeds showed sharp increases at stages 4 and 5 (Fig. 1). In contrast, the oil content in caruncles remained low at stage 4 but increased significantly at stage 5, reaching 40% on average at maturation, although it was lower than the oil content in seeds at the same stage. At stage 5, caruncle had 10.6% total soluble sugar content by dry weight, compared to 2.8% in developing seed at the same stage (Additional file 1: Table S1). Caruncle also accumulated low amount of starch, while seed had very low starch level (Additional file 1: Table S1).

### The distinct fatty acid profile of caruncles

**Fig. 2:**
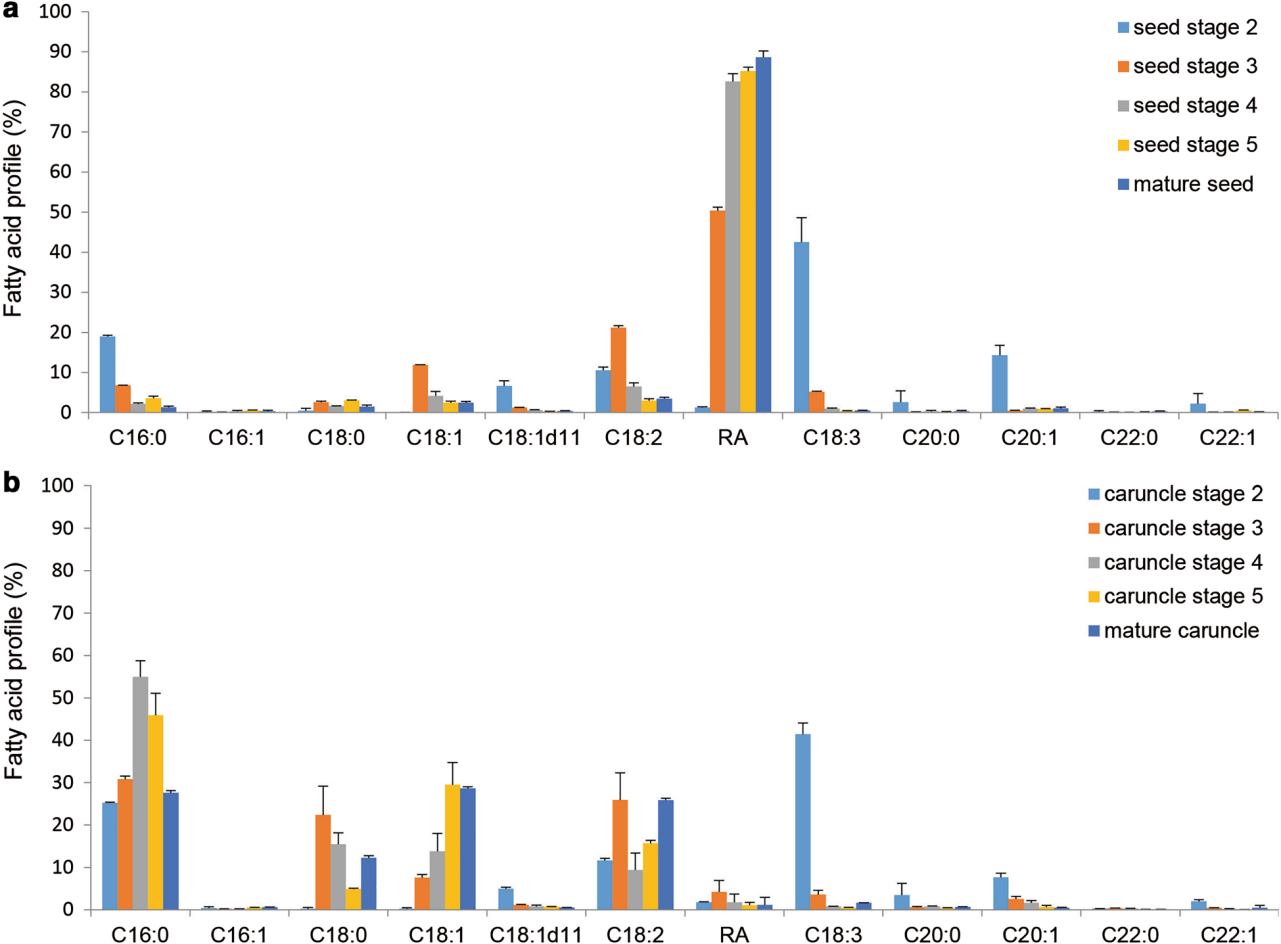
Fatty acid composition of total lipid in castor seeds and the caruncles during seed development. **a** Seed including endosperm and embryo. **b** The caruncle. *n* = 6. S2–S6 and C2–C6 are seeds and caruncles at stages 2–6

The unsynchronized oil accumulation between seeds and caruncles prompted us to investigate whether the oil in caruncles was synthesized independently or originated from the seed, considering the physical connection of these two types of tissues. The fatty acid composition of total lipids in seeds and caruncles during fruit development was first compared by gas chromatography (GC) analysis. In developing seeds, the major fatty acid at the early stages, i.e., stage 2 of development was C18:3, but RA became dominant from stage 3 and reached 89% in mature seed oil composition, suggesting that rapid biosynthesis of RA occurred at the late development stage of castor bean seeds (Fig. 2a). In caruncles, although C18:3 was also the major fatty acid at stage 2, the major fatty acids by the late development stages were C16:0, C18:1 and C18:2, each of which comprised about 30% of total fatty acids; while C18:3 was only less than 2% of total fatty acids, at maturity (Fig. 2b). The dominant C18:3 in both seeds and caruncles at stage 2 when the overall oil content was very low could be attributable to membrane lipids, rather than TAGs. In contrast to its high-level accumulation in seeds, RA was barely detectable in caruncles. The striking difference in fatty acid profiles between caruncles and seeds might suggest that caruncle oil was synthesized independent of seed, and transportation of TAGs or lipid intermediates from seeds was unlikely.

### Independent fatty acid modification and TAG synthesis in caruncles

**Fig. 3:**
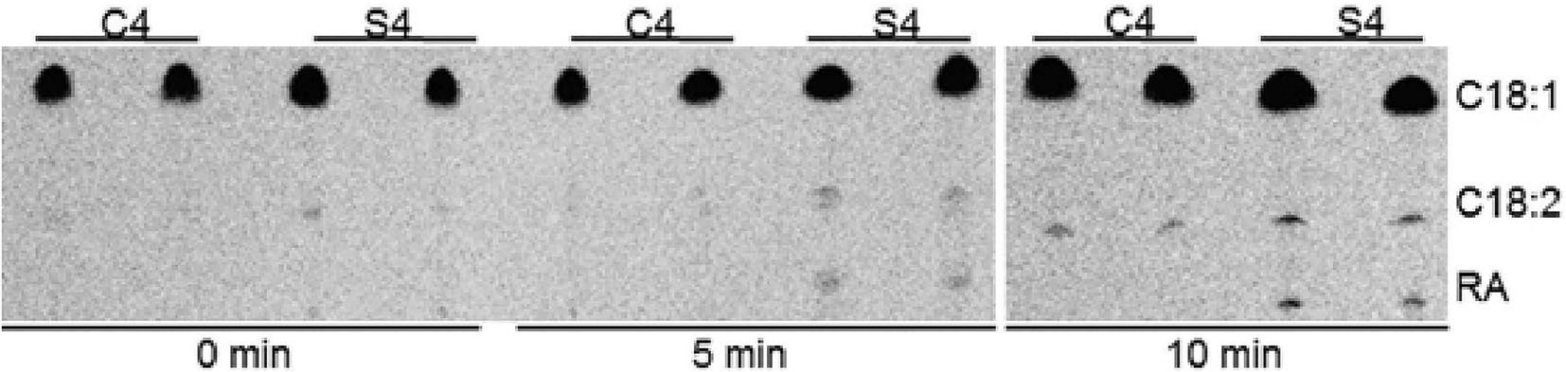
Desaturation and hydroxylation products from fed [^14^C]-C18:1-CoA in in vitro assay. Duplicate assay was shown for 0, 5 and 10 min with microsomal proteins from stage 4 seed (S4) or the caruncle (C4)

The ability of caruncles to modify fatty acids and synthesize TAG was investigated. Microsomal proteins from the developing caruncles at stage 4 were prepared for desaturation and hydroxylation assays using labeled [^14^C]-C18:1-CoA substrate and compared to seed microsomal proteins at the same stage. As shown in Fig. 3, after incubation for 10 min, there was an accumulation of the desaturated C18:2 product in caruncles, while both C18:2 and the hydroxylated RA product were detected in seed microsomal proteins. Extended incubation for 20 min did not result in obvious RA synthesis in caruncles (data not shown). This result indicates that caruncles have no or very limited capability for fatty acid ∆12-hydroxylation, and possibly the general fatty acid modification pathway acts independent of the seed.

**Fig. 4:**
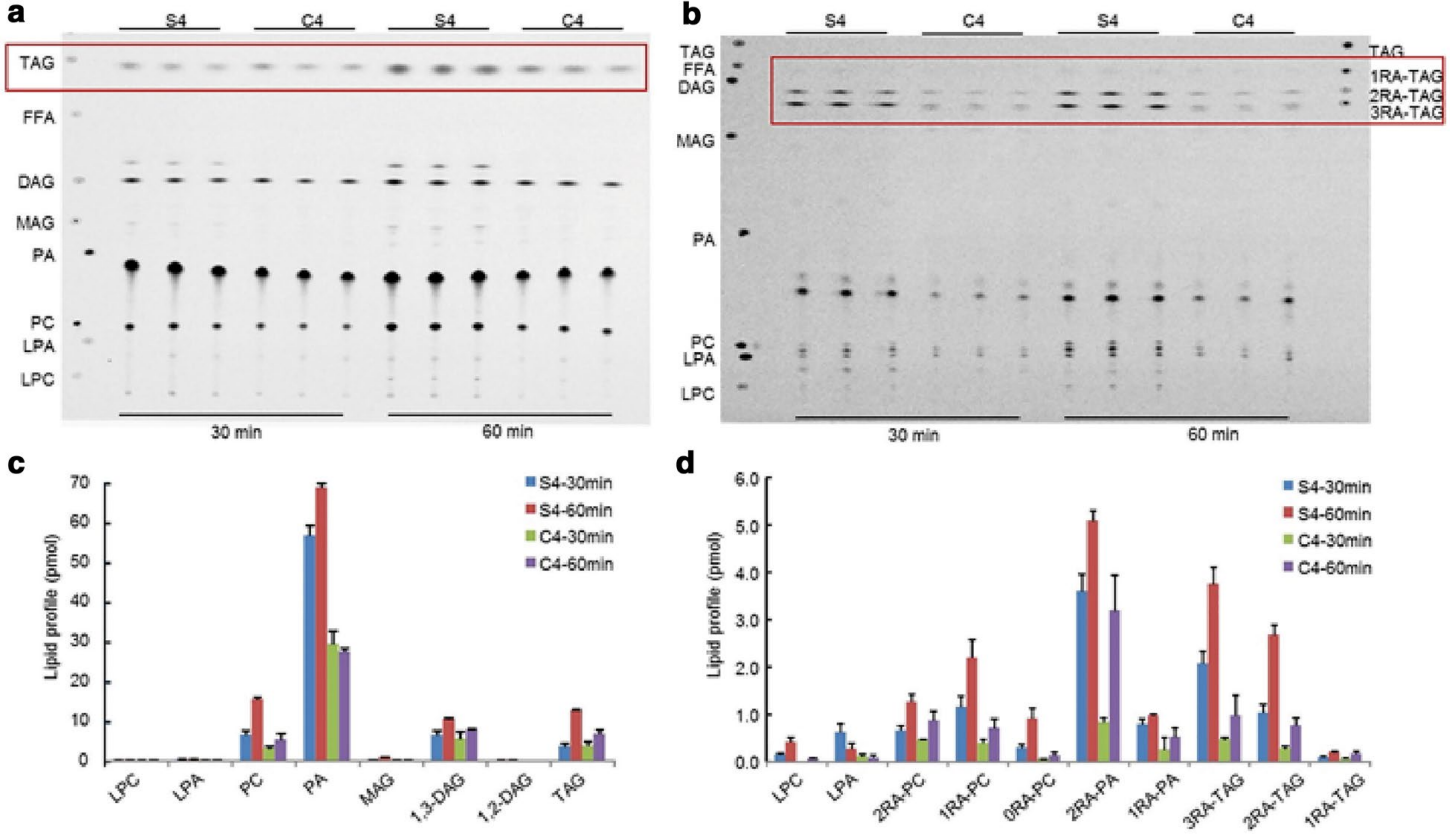
*In vitro* triacylglycerol (TAG) synthesis from stage 4 seed (S4) and the caruncle (C4) microsomal proteins, incubated with (**a**, **c**) [^14^C]-G3P and oleoyl-CoA or (**b**, **d**) [^14^C]-G3P and ricinoleoyl-CoA. Error bars show the standard deviations from triplicate assays for 30 or 60 min. *DAG* diacylglycerol, *FFA* free fatty acid, *G3P* glycerol-3-phosphate, *GPAT* glycerol-3-phosphate acyltransferase, *LPA* lysophosphatidic acid, *LPC* lysophosphatidylcholine, *LPCAT* lysophosphatidylcholine acyltransferase, *MAG* monoacylglycerol, *PA* phosphatidic acid, *PC* phosphatidylcholine, *RA* ricinoleic acid, *TAG* triacylglycerol

The same microsomal proteins were then used for a TAG synthesis assay. When microsomal proteins were incubated with [^14^C]-glycerol-3-phosphate (G3P) and oleoyl-CoA, both seed and caruncle tissues produced TAG (Fig. 4a), although the overall activity of the caruncle proteins was lower than that of the seed proteins (Table 1). The activities of glycerol-3-phosphate acyltransferase (GPAT) and lysophosphatidic acid acyltransferase (LPAAT) in both tissues were high as suggested by the significant PA accumulation. Similar to the desaturation activity, the overall activity of the Kennedy pathway in the seed microsomal proteins was higher than that in the caruncles. When fed with ricinoleoyl-CoA, the seed proteins synthesized lower amounts of TAG in comparison to feeding with oleoyl-CoA (Fig. 4b, Table 2). Compared to seed proteins, the caruncle proteins synthesized much less TAG from oleoyl-CoA (about 50%) or ricinoleoyl-CoA (about 30%). These results again indicate that caruncles have the capability of independent TAG synthesis, with a different acyl-CoA preference, from the seed.

**Table 1:** G-3-P feeding of microsomal protein from seed or the caruncle at stage 4 with oleoyl-CoA

Samples	Time (min)	Percentage of total (%)									
		LPA	PA	LPC	PC	MAG	1,2-DAG	1,3-DAG	TAG	Unknown	Total pmol
Seed	30	0.4 ± 0.1	75.8 ± 2.5	0.1 ± 0.0	8.5 ± 1.1	0.4 ± 0.1	8.6 ± 1.1	1.3 ± 0.2	4.8 ± 0.7	0.1 ± 0.0	3.6 ± 0.0
	60	0.3 ± 0.0	61.7 ± 1.0	0.2 ± 0.0	13.8 ± 0.7	0.7 ± 0.1	9.3 ± 0.3	2.4 ± 0.1	11.48 ± 0.2	0.2 ± 0.0	12.9 ± 0.0
Caruncle	30	0.4 ± 0.1	70.0 ± 3.3	0.1 ± 0.0	7.3 ± 0.6	0.2 ± 0.1	13.1 ± 1.8	NG	9.0 ± 1.0	0.0 ± 0.0	3.8 ± 0.0
	60	0.3 ± 0.1	57.7 ± 0.9	0.1 ± 0.1	11.3 ± 1.6	0.2 ± 0.0	16.2 ± 0.3	NG	14.0 ± 1.1	0.1 ± 0.0	6.7 ± 0.0

**Table 2:** G-3-P feeding of microsomal protein from seed or the caruncle at stage 4 with ricinoleoyl-CoA

Samples	Time (min)	Percentage of total (%)										Total pmol
		LPA	1RA-PA	2RA-PA	LPC	0RA-PC	1RA-PC	2RA-PC	1RA-TAG	2RA-TAG	3RA-TAG	
Seed	30	5.9 ± 0.9	7.5 ± 0.4	34.6 ± 1.3	1.4 ± 0.3	2.8 ± 0.3	11.0 ± 0.6	6.3 ± 0.8	0.8 ± 0.4	9.9 ± 0.3	19.8 ± 0.4	3.2 ± 0.2
	60	1.5 ± 0.6	5.4 ± 0.8	28.5 ± 4.3	2.3 ± 0.3	5.2 ± 0.4	12.4 ± 2.2	7.1 ± 0.7	1.1 ± 0.0	15.2 ± 1.6	21.3 ± 0.9	6.6 ± 0.3
Caruncle	30	3.7 ± 1.5	9.0 ± 1.5	28.5 ± 3.4	0.0 ± 0.4	1.9 ± 0.2	13.5 ± 2.5	16.0 ± 0.9	2.1 ± 0.3	9.6 ± 1.7	16.0 ± 1.2	0.8 ± 0.1
	60	0.9 ± 0.5	7.1 ± 0.6	41.5 ± 5.8	0.5 ± 1.2	2.0 ± 1.1	9.9 ± 1.3	12.1 ± 1.8	0.0 ± 0.3	10.8 ± 2.1	13.0 ± 0.4	1.9 ± 0.3

### Dissecting the genes involved in fatty acid and TAG synthesis in caruncles

The differences in oil content, fatty acid profile, fatty acid desaturation activity and TAG synthesis between caruncles and seeds prompted us to propose that the gene set used for TAG synthesis in caruncles might be different from that in the seed. It was, therefore, interesting to compare the gene expression patterns of caruncles and seeds to identify the gene set that contributes to oil accumulation in non-seed tissue. Deep sequencing was performed in triplicate for the RNA isolated from seeds and caruncles at stage 4 of seed development. A total of 163,972,525 and 147,592,710 clean reads were obtained from seeds and caruncles, respectively. A list of differentially expressed genes involved in fatty acid metabolism and TAG synthesis was identified in caruncles compared to seeds (Additional file 2: Table S2), based on the annotation of the published castor genome sequence [24].

**Fig. 5:**
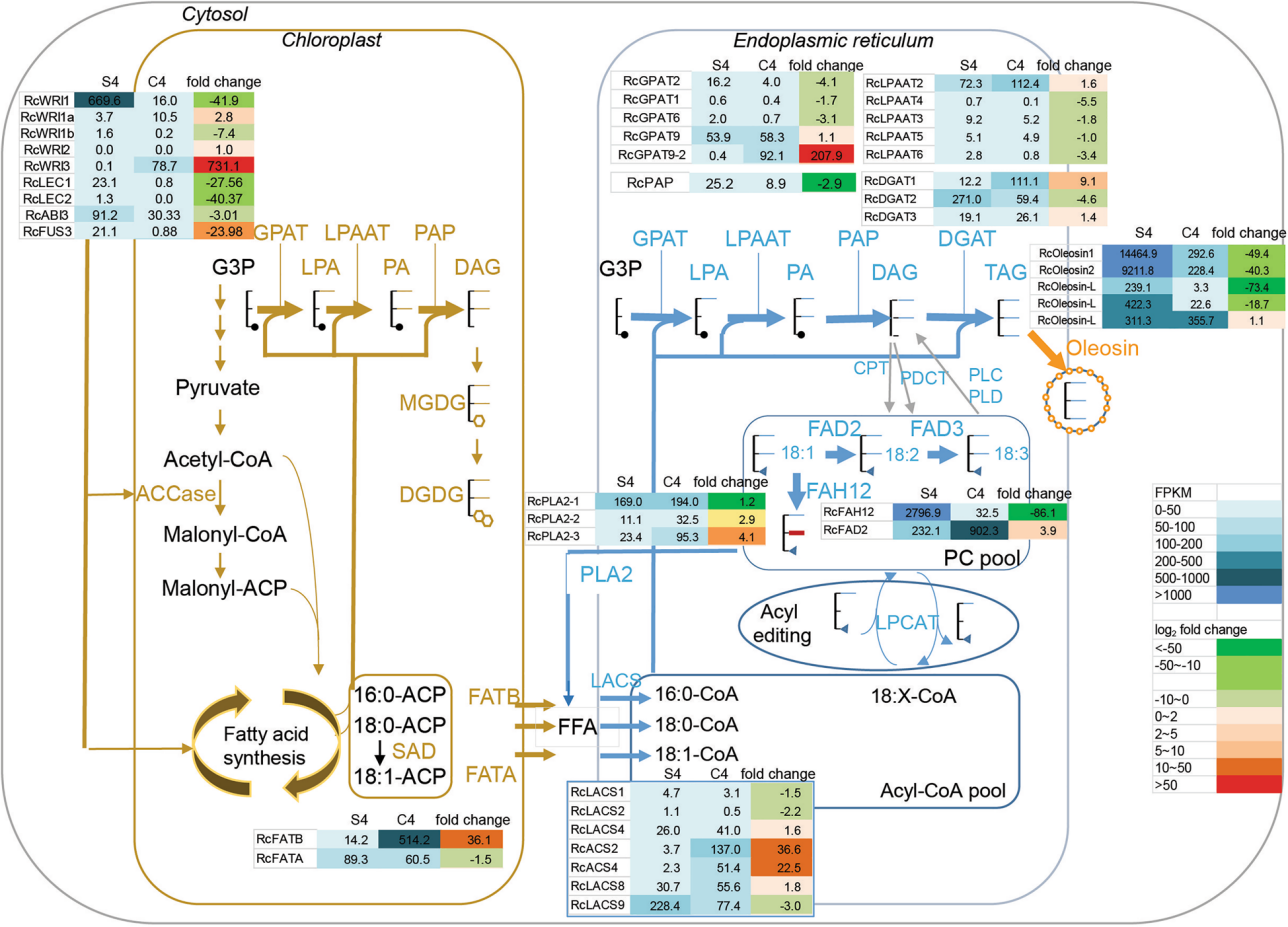
Transcriptome analysis of lipid synthesis genes in developing seed and the caruncle. The expression levels of key classes of genes involved in fatty acid and TAG synthesis are listed in fragments per kilobase per million mapped reads (FPKM) with fold change of expression level of the caruncle vs. seed (log2). S4, seed stage 4; C4, the caruncle stage 4. The FPKM and fold change values are color coded with the scale shown in inlet. *ACCase* acetyl-CoA carboxylase, *ACP* acyl carrier protein, *DAG* diacylglycerol, *CoA* coenzyme A, *CPT* cholinephosphotransferase, *DAG* diacylglycerol, *DGAT* acyl-CoA:diacylglycerol acyltransferase, *DGDG* digalactosyldiacylglycerol, *FAD2* fatty acid desaturase 2 (Δ12-desaturase), *FAD3* fatty acid desaturase 3 (Δ15-desaturase), *FAH12* Δ12-hydroxylase, *FFA* free fatty acid, *FATA or FATB* acyl acyl-carrier-protein thioesterase A or B, *G3P* glycerol-3-phosphate, *GPAT* glycerol-3-phosphate acyltransferase, *LACS* long-chain acyl-CoA synthetase, *MGDG* monogalactosyldiacylglycerol, *LPA* lysophosphatidic acid, *LPAAT* lysophosphatidic acid acyltransferase, *LPC* lysophosphatidylcholine, *LPCAT* lysophosphatidylcholine acyltransferase, *PA* phosphatidic acid, *PAP* phosphatidic acid phosphatase, *PC* phosphatidylcholine, *PDAT* phospholipid:diacylglycerol acyltransferase, *PDCT* phosphatidylcholine:diacylglycerol cholinephosphotransferase, *PLA2* phospholipase A2, *PLC* phospholipase C, *PLD* phospholipase D, *SAD* stearoyl-ACP desaturase, *WRI* WRINKLED (transcription factor), *TAG* triacylglycerol

Among the genes for transcriptional factors known to be involved in fatty acid synthesis, *RcWRI1* (30069.m000440) was the most highly expressed gene in seeds but its expression in caruncles was relatively low (Fig. 5). In contrast, the expression levels of *RcWRI1*-*like* (*RcWRI1a*, 30006.m000282) and *RcWRI3* (30131.m006896) in caruncles were higher than in seeds. *RcWRI3* was of interest as it is the most highly expressed transcriptional factor in caruncles and it was highly up-regulated (731-fold increase) compared to seed. The expression levels of most genes that are involved in fatty acid synthesis were not dramatically increased in caruncles. A second gene that showed higher expression levels in caruncles than in seeds was acyl-acyl carrier protein (acyl-ACP) thioesterase B (*RcFATB*, 29848.m004677), which might release saturated free fatty acid C16:0 from acyl-ACP and enable its export from plastids. *RcFATB* was up-regulated 36-fold; whereas another acyl-ACP thioesterase gene *RcFATA* (30217.m000262) was down-regulated in caruncles relative to seeds (Fig. 5). Another acyl-ACP thioesterase-like gene *RcFATB*-*L* (30147.m014468), had rather low expression in both seeds and caruncles (Additional file 2: Table S2). Following the action of acyl-ACP thioesterase, free fatty acids would then be converted into acyl-CoA by the action of acyl-CoA synthetase (ACS) or long-chain acyl-CoA synthetase (LACS) in the cytosol. Among seven *ACS*-like genes, the most highly expressed gene in caruncles was *RcACS2* (29844.m003365), while the expression of *RcLACS9* (29908.m006186) was the highest in seeds (Additional file 2: Table S2). The expression of *RcACS2* and *RcACS4* (30128.m008777) was higher in caruncles than in seeds, with 36-fold and 22-fold increase, respectively. In contrast, the expression of *RcLACS9* was lower in caruncles than in seeds.

A subset of genes for TAG synthesis and storage was also identified as up-regulated in caruncles compared to seeds. Three sequentially functioning acyltransferases are involved in the acylation of G3P from acyl-CoA donors for TAG synthesis, via the acyl-CoA-dependent Kennedy pathway. These include genes encoding for GPAT, lysophosphatidic LPAAT and diacylglycerol acyltransferase (DGAT). The Kennedy pathway genes that showed high expression levels in caruncles were *RcGPAT9*-*2* (29908.m005967) and *RcDGAT1* (29912.m005373). Among the *GPAT* genes, *GPAT9* (30122.m000357) had similar levels of expression in caruncles and in seeds, but *GPAT9*-*2* had the highest expression level among the *GPAT* genes in caruncles with a 207-fold increase compared to seeds (Additional file 2: Table S2, Fig. 5). There was no significant difference in expression levels of LPAAT family members, with *RcLPAAT2* (27810.m000646) being the most highly expressed gene in both seeds and caruncles and with slightly higher expression in caruncles than in seeds (1.6-fold) (Additional file 2: Table S2, Fig. 5). Three genes that encode for DGATs which catalyze the last and the rate-limiting enzymatic step of the Kennedy pathway were identified. *RcDGAT1* (29912.m005373) showed higher expression in caruncles with a ninefold increase relative to seeds, while *RcDGAT2* (29682.m000581) showed higher expression in seeds than in caruncles. The expression of *RcDGAT3* (29889.m003411) which encodes a soluble DGAT was low yet at similar levels in both caruncles and seeds (Additional file 2: Table S2, Fig. 5). Oleosins are integral oil body proteins that play key roles in TAG packaging and maintenance of oil body integration during seed maturity. Two genes encoding for oleosin 1 (29917.m001992) and oleosin 2 (30147.m014333) were expressed at much lower levels in caruncles than in seeds. The expression levels of three other oleosin-like protein (RcOleosin-L) genes (30147.m013891, 29794.m003372 and 30147.m998728) were either lower in caruncles or similar to seeds. Each of three putative lipid droplet-associated proteins (RcLDAPs, 30128.m008823, 30129.m000374 and 29929.m004653) showed similar expression levels between seeds and caruncles. Other genes involved in lipid metabolism showing higher expression in caruncles than in seeds included genes for fatty acid Δ12-desatursae RcFAD2 (29613.m000358, fourfold), monoacylglycerol lipase (29113.m000030), and lipid transfer protein (27996.m000148) (Additional file 2: Table S2). The relative expression of the above-mentioned genes between caruncles and seeds was verified by Real-Time Quantitative Reverse Transcriptase PCR (qRT-PCR, see Additional file 3: Table S3).

### Transient expression of selected genes for enhancing TAG accumulation in leaf

**Fig. 6:**
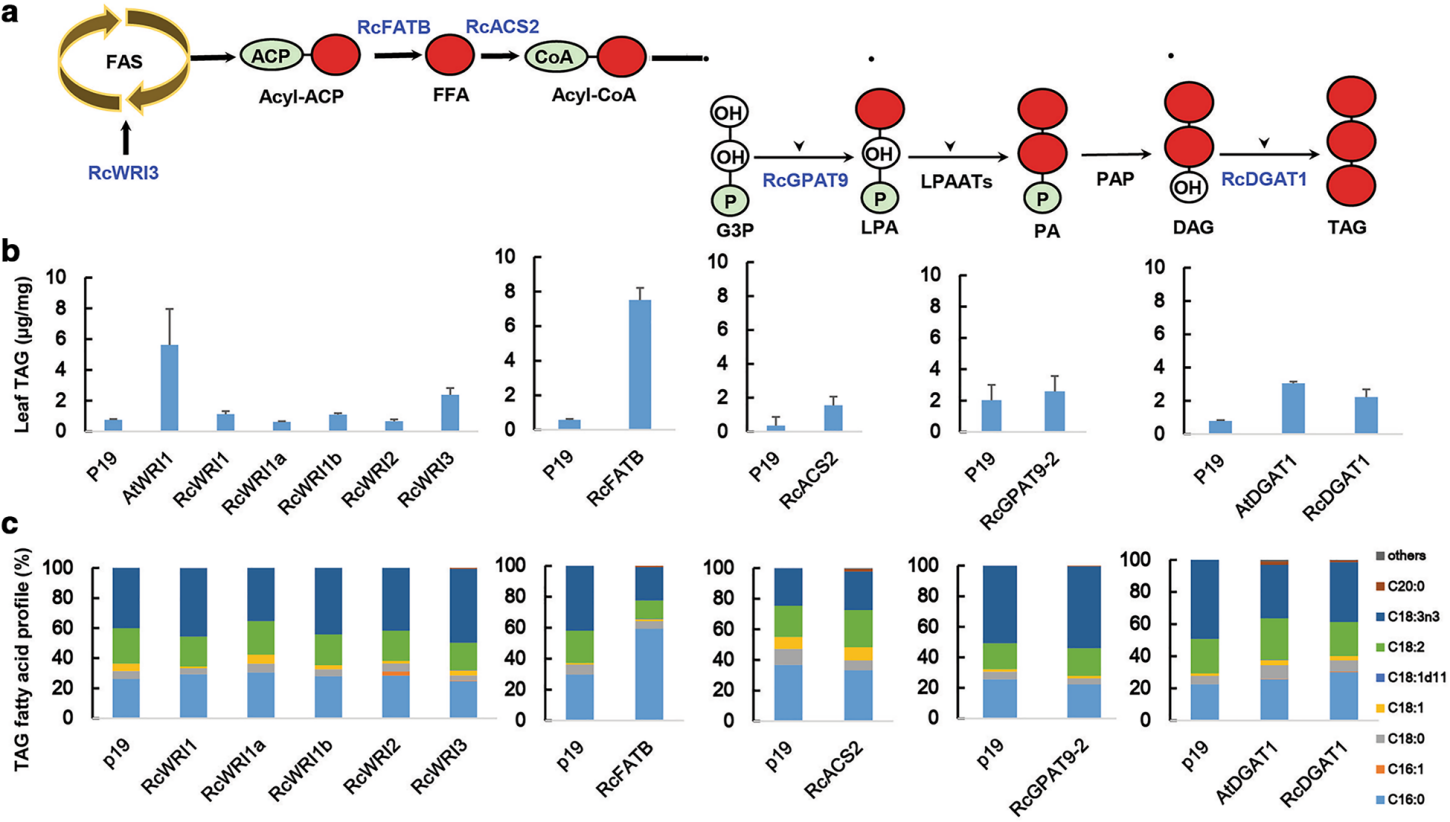
Transient overexpression of the selected castor caruncle genes in *Nicotiana benthamiana* leaves. Graphic abstract representing up-regulated genes involved in fatty acid and TAG synthesis from the castor caruncle in blue (**a**). TAG accumulation (mg TAG per mg DCW) (**b**) and TAG fatty acid profile (**c**) in *N. benthamiana* leaves expressing the selected genes. Error bars denote standard deviation with *n *= 9. *ACP* acyl carrier protein, *ACS* acyl-CoA synthetase, *DAG* diacylglycerol, *DGAT* acyl-CoA:diacylglycerol acyltransferase, *FFA* free fatty acid, *FATB* acyl acyl-carrier-protein thioesterase B, *G3P* glycerol-3-phosphate, *GPAT* glycerol-3-phosphate acyltransferase, *LPA* lysophosphatidic acid, *LPAAT* lysophosphatidic acid acyltransferase, *PA* phosphatidic acid, *PAP* phosphatidic acid phosphatase, *WRI* WRINKLED (transcription factor), *TAG* triacylglycerol

Based on the RNA sequencing, a set of genes that are involved in major steps of fatty acid or TAG biosynthesis was identified based on their higher expression in caruncles than in seeds. The potential functional role of these genes in increasing oil accumulation or altering fatty acid profile in non-seed plant tissues, such as leaves, was investigated by transient expression in *N. benthamiana*, either individually or in combination (Fig. 6).

We first compared the TAG levels in *N. benthamiana* leaves expressing different RcWRI transcription factors, using *Arabidopsis* AtWRI1 as a control. In addition to the three previously characterized castor *RcWRI* genes [25], two other *RcWRI1*-like genes (30006.m000282 and 29822.m003477, designated as *RcWRI1a* and *RcWRI1b*) were identified based on sequence homology. Expression of RcWRI3 led to an increase in TAG content in leaves compared to the p19 control, in a manner similar to, although not as effective as, AtWRI1 (Fig. 6b). The expression of RcWRI1 in leaves led to only a slight increase in TAG content, even though it was identified as the most highly expressed *WRI* gene in seeds. The expression of other WRI1-like or WRI2 transcriptional factors in leaves resulted in no significant change in the amount of TAG. Overexpression of the acyl-ACP thioesterase gene, *RcFATB*, resulted in a 13-fold increase in TAG content compared to the P19 control (Fig. 6b). In addition, there was a significant change in the fatty acid profile in the TAG fraction, with the C16:0 proportion raised from 26.2 to 49.0% (Fig. 6c). Due to the substantial increase in total TAG content in RcFATB-expressing tissues, the absolute amounts of polyunsaturated fatty acids C18:2 and C18:3 were also increased from 0.12 and 0.24 µg per mg of dry leaves in control to 0.89 µg and 1.61 µg per mg of dry leaves that expressed RcFATB, respectively, despite the reduction in their relative proportions in total TAGs (Fig. 6b). The enhanced accumulation of C16:0 in leaf TAGs due to expression of RcFATB may explain the relatively higher proportion of C16:0 in caruncles than in seeds. This is also consistent with a 36-fold higher expression of RcFATB in caruncles than in seeds. Being the most highly expressed gene in caruncles, *RcACS2* (29844.m003365) showed the strongest upregulation in caruncles compared to seeds and relative to all other *ACS* genes examined (Fig. 5). Overexpression of RcACS2 resulted in an increase in TAG content from 0.35 µg/mg in the control to 1.55 µg per mg of dry leaves, with a slightly reduced proportion of C16:0 (Fig. 6b). On the other hand, overexpression of the *RcACS4* (30128.m008777) or *RcLACS9* (29908.m006186) genes did not result in obvious TAG increases (data not shown). Overexpression of the *RcGPAT9* gene did not result in statistic difference of TAG (data not shown), while the *RcGPAT9*-*2* gene resulted in only a slight increase in TAG. Overexpression of the *RcDGAT1* gene resulted in a significant increase in TAG accumulation and a slightly increased proportion of C18:1, but the increase in the amount of TAG was not as high as overexpression of the Arabidopsis *AtDAGT1* gene.

### Additive effect of expressing the selected genes for increased leaf TAG accumulation

**Fig. 7:**
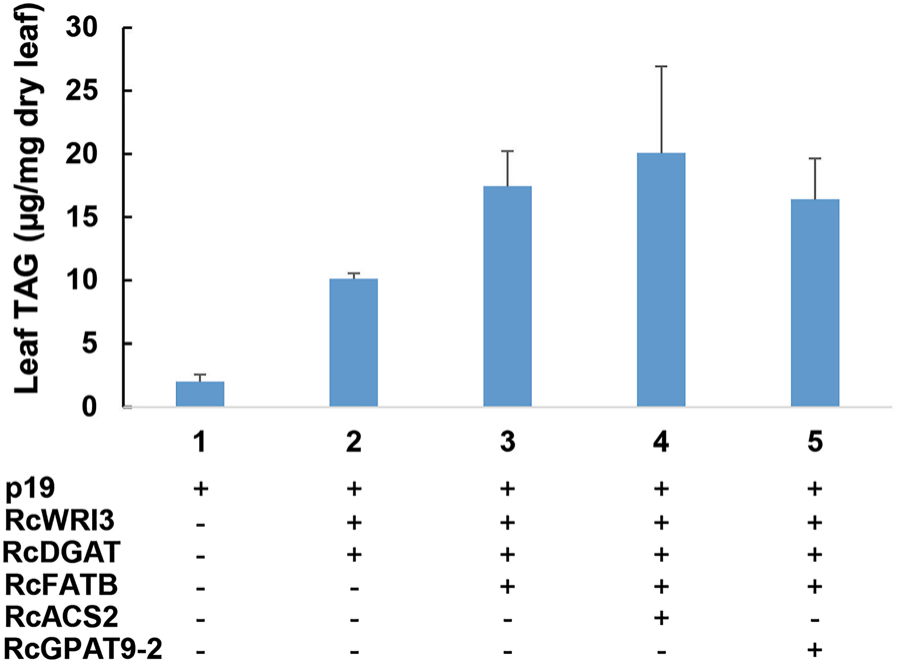
Increased leaf TAG accumulation by combined expression of the selected genes. Symbols “+” or “−” indicate the gene included or excluded in the assays 1 to 5. Error bars denote standard deviation with *n *= 3

Because most of the selected genes tested with higher expression levels in caruncles resulted in significant or slight increases in TAG accumulation in the transient assay, we further overexpressed these genes in combination to evaluate their compound effects on TAG production. The *RcWRI3* and *RcDGAT1* combination repeatedly produced further increased amounts of TAGs compared to the expression of the individual genes alone. Co-expression of *RcWRI3* and *RcDGAT1* resulted in 11.0 µg TAGs per mg of dry leaves, compared to 2.0 µg TAGs from the control in the same experiment (Fig. 7). Step-wise stacking of additional caruncle genes in combination with *RcWRI3* and *RcDGAT1* led to further increased TAG synthesis. The addition of *RcFATB* increased the TAG content up to 17.4 µg per mg of dry leaves. *RcACS2* expression on top of *RcWRI3*, *RcDGAT1*, and *RcFATB* further increased the TAG amount to 20.1 µg per mg of dry leaves, or a tenfold increase compared to the p19 control; whereas there was no further increase with additional expression of *RcDGAT9*-*2*. When *RcFATB* was included in these combinations, the enhanced C16:0 level was observed (Additional file 4: Table S4). These results suggested that the cooperation of these key genes in caruncles led to the high oil accumulation. Such gene sets are a likely future resource for metabolic engineering of oil production in non-seed tissues like leaves.

## Discussion

We show here that caruncle which is associated to but outside of seed coat had clear differences in the contents of oil, total soluble sugars and starch and fatty acid profile from seed. Biochemical evidence confirmed that caruncle had distinct fatty acid modification and TAG synthesis activities compared to seed. Although the starch content was low in caruncle, it was much higher than in seed. In sharp contrast to seed, caruncle also contained a double-digit level of total soluble sugars which could provide the precursors for fatty acid synthesis. Earlier cytological work has also shown that caruncle consists of epidermal and parenchyma cells which accumulate oil [17]. Comparative transcriptome analysis of developing seed and caruncle also showed their distinct gene expression patterns. These all supported the hypothesis that caruncle was an oil-rich non-seed tissue that could actively synthesize TAG independent of seed.

Plant oils derived from oil-rich non-seed tissues have attracted increasing attention in recent years. Biotechnology is a promising approach to generate enhanced levels of oil in high biomass plant vegetative tissues. Most of the genes used so far by metabolic engineers to increase oil content in non-seed tissues have been sourced from oilseed tissues. Since the discovery of LDAPs from oil-rich non-seed tissues [26], it is suggested that such genes with a non-seed origin may be more likely to enhance the oil content in non-seed tissues with minimal pleiotropic effects on plant growth and agronomic traits.

The differential expression of key genes in caruncle and seed, and its putative impacts on fatty acid and TAG biosynthesis are summarized in Additional file 5: Figure S1. Two sets of key genes which might play a key role on the accumulation of major fatty acids in TAG of caruncle or seed are highlighted in blue or in dark red, respectively. The gene set in seed (dark red) could have major contribution for high accumulation of RA in castor bean seed. The WRI1 transcription factor initiates concomitant upregulation of genes involved in fatty acid production as seeds reach maturity [27], and it binds to the promoter sequences of genes involved in the late glycolytic and fatty acid biosynthetic pathways [28]. Four *WRIs* including *WRI1*, *WRI2*, *WRI3* and *WRI4* have been isolated from *Arabidopsis* [29]. Only WRI1 is proven to activate fatty acid biosynthesis leading to TAG production in seeds. WRI1, WRI3 and WRI4 are active in floral tissues to provide acyl precursors for cutin biosynthesis and prevent floral organ fusions and subsequent sterility [29]. In contrast, WRI2 does not activate the transcription of fatty acid biosynthetic genes such as *BCCP2* (At5g15530) or *PDH*-*E1a* (At1g01090) and its function might not be related to fatty acid biosynthesis [29]. Three RcWRI1 homologs have also been identified from castor [25]. The castor *RcWRI1* gene is expressed at high levels in developing seed and plays an important role in the regulation of seed-specific expression of FAS genes [25]. Transcriptome analysis has revealed that the transcript abundance of *RcWRI3* in leaves and flower was 10.3 fragments per kilobase per million mapped reads (FPKM) and 63.8 FPKM, respectively, but was not discernible in endosperm [24]. Our sequencing results showed 0.1 and 78.7 FPKM of *RcWRI3* in seeds and caruncles, respectively, confirming the high expression of *RcWRI3* in non-seed tissue. *RcWRI3* was the most highly expressed *WRI* gene in caruncle and its transient expression in *N. benthamiana* leaves gave rise to the highest TAG accumulation among the five *RcWRIs*. In contrast, *RcWRI1* that had very high expression levels in seeds at 669.6 FPKM resulted in only moderate TAG accumulation in leaves. Considering that the castor oil is mainly accumulated in endosperm rather than embryo, RcWRI1 might have different functional pattern compared to Arabidopsis AtWRI1, which has been shown to be effective in increasing TAG accumulation in leaf [30]. RcWRI3 was the key transcription factor for TAG accumulation in the castor caruncle.

Acyl-ACP thioesterases are the key enzymes that release fatty acids from acyl-ACP to free fatty acids, and their differential expression determines the type of fatty acids that are exported to the cytosol and hence become available for re-esterification into acyl-CoA by acyl-CoA synthetase (ACS) enzymes, and further incorporation into glycerolipids in endoplasmic reticulum (ER). FATA shows high substrate specificity towards C18:1-ACP, while FATB prefers C16:0-ACP [31]. At least three acyl-ACP thioesterase genes have been identified in castor bean, including *RcFATA* (3027.m000262), *RcFATB* (29848.m004677) and *RcFATB*-*L* (30147.m014468). *RcFATB1* was the most highly expressed gene in the caruncle but the expression of *RcFATB2* was rather low in both seed and the caruncle tissues. *RcFATA* was the second most highly expressed gene in the caruncle, with slightly lower expression level than in seeds. The expression of *RcFATB* in *Escherichia coli* showed high catalytic efficiency with both oleoyl-ACP and palmitoyl-ACP [32]. Our recent work also showed the preference of RcFATB towards C14:0, and increased TAGs due to RcFATB activity in *E. coli* [33]. Overexpression of *RcFATB* alone in leaves resulted in significant increases in TAG accumulation, with strong preference for C16:0 accumulation among the TAGs. Additional expression of *RcFATB* also enhanced the additive effect of RcWRI3/RcDGAT1 on leaf TAG accumulation. Our work agrees with the previously reported TAG enhancement by overexpressing *AtFATB* [34].

Because acyl-CoA is the acyl donor for multiple steps of the Kennedy pathway of TAG synthesis, ACS plays an important role in the metabolism of free fatty acids into storage lipid. Seven ACS-like sequences have been identified from the castor genome [24]. *RcACS2* (29844.m003365) was identical to the previously characterized *RcACS2* gene (DQ300358), which restores growth of a yeast mutant strain deficient in ACS [35]. RcACS2 is most closely related to AtLACS6 (At3g05970) among the nine Arabidopsis LACS enzymes (data not shown). AtLACS6 is targeted to peroxisomes [36], but it was not able to complement the YB525 yeast mutant, which is unable to produce the necessary levels of acyl-CoA, in spite of its confirmed LACS activity [37]. RcACS2 is a likely peroxisomal ACS isoform with a peroxisomal targeting sequence [38]. In contrast to its Arabidopsis ortholog, RcACS2 is able to complement YB525 [35]. *RcACS2* was highly expressed in seeds during germination [24, 35], likely functioning to activate fatty acids to acyl-CoA for degradation through β-oxidation. RcACS2 has been shown to have higher preference for hydroxy fatty acid [35]. In the current work, overexpression of *RcACS2* did enhance the TAG accumulation in leaves, suggesting the acyl-CoAs synthesized by RcACS2 might also be available for TAG synthesis.

Multiple GPATs have been identified from castor bean by BLAST analysis [23], with RcGPAT1 (30068.m002660) being predicted to be located in plastids. Other cytosol-localized GPATs are mostly involved in the synthesis of cutin and suberin [39]. Castor GPAT9 (30122.m000357), which is localized in the ER, has the highest FPKM value amongst *GPATs* in endosperm [24] potentially contributing to ricinoleic acid-containing TAG synthesis. Another RcGPAT sequence (29908.m005967) which was also designated as RcGPAT9 by Cagliari et al. [23] was the most highly expressed member in caruncles, over 207-fold higher than in seeds. Its low expression in seeds has been reported [24]. To make a distinction from the RcGPAT9 reported by Brown et al. [24], here we designated the sequence as *RcGPAT9*-*2*, as reported by Cagliari et al. [23]. In the subsequent leaf expression test, overexpression of RcGPAT9-2 showed slightly increased TAG accumulation.

Three DGAT enzymes have been identified in castor, including a membrane-bound type 1 DGAT (RcDGAT1), a type 2 DGAT (RcDGAT2), and a soluble RcDGAT3. RcDGAT2 is considered to have preference for ricinoleoyl-CoA as substrate in TAG synthesis [40]. The expression level of *RcDGAT2* in seeds was much higher than in caruncle, being consistent with the high proportion of RA accumulation in seed oil. In contrast, *RcDGAT1* was the most highly expressed *DGAT* gene in caruncle with significantly higher expression than in seeds. Considering the difference in the fatty acid profile between the caruncle and seed TAGs, RcDGAT1 might contribute to the TAG synthesis in caruncles with minimal RA accumulation. Our results did show that the overexpression of *RcDGAT1* led to enhanced accumulation of TAGs in leaves. *RcWRI3* and *RcDGAT1* combination repeatedly produced further increased amount of TAG compared to the expression of the individual gene alone. This result is consistent with the previous report that the combined expression of *AtWRI1* and *AtDGAT1* on TAG enhancement in tobacco leaves [30]. The combined expression with some other above-mentioned genes in leaf had further increased the TAG level, with the best gene combination being *RcWRI3*, *RcDGAT1*, *RcFATB* and *RcACS2*. This study, thus, revealed an additive effect of a set of non-seed expressed genes on TAG accumulation in leaves, which may serve as a useful tool for choosing gene combinations for higher TAG production in non-seed plant tissues or microbes.

## Conclusions

Castor non-seed tissue caruncle independently synthesizes and accumulates up to 40% TAG, with a highly contrasting fatty acid composition when compared to the seed oil. Several distinct genes or transcriptional factors responsible for fatty acid synthesis and TAG assembly in the caruncle are identified. Combinational expression of selected genes resulted in increased TAG accumulation in leaves. It provides the possibility of exploiting caruncle gene set to engineer oil production in non-seed tissues or microbes.

## Methods

### Plant materials

Developing castor seeds were collected and grouped into different development stages after removing the seed coat, based on morphologic characteristics, such as size, color and texture of the seed according to Cagliari et al. [23]. The attached caruncles were grouped into same stage. The castor seed and the caruncle were frozen immediately in liquid nitrogen after harvest and stored at − 80 °C until use.

### Analysis of lipid content and fatty acid profile in castor seed and the caruncle

Total lipids were extracted from freeze-dried seed and the caruncle using chloroform/methanol/KCl method [41]. TAG was fractionated by thin layer chromatography (TLC) using silica gel 60 (Merck) developed with hexane:diethylether:acetic acid (70:30:1, v/v/v) and visualized by spraying Primuline (Sigma, 5 mg/100 mL 80% acetone). Silica with TAG fraction was recovered under UV illumination. Fatty acid methyl esters (FAME) of TAG or total lipids were produced by methanolic/HCl method [41]. The oil contents in seed and the caruncle were measured with MQC-23 nuclear magnetic resonance (NMR) Analyzer (Oxford Instruments), hr. Calibration samples were prepared with dried Kimwipes papers with certain amount of purchased pure castor oil in 10-mm-diameter NMR test tubes, to generate a range of oil content based on the weights of paper and oil. The calibration samples were sealed with parafilm and maintained at 40 °C heating block minimal 1 h before using to allow oil distribute uniformly in the tissue. The calibration samples were measured three times with 0.55-Tesla magnet and 10-mm-diameter probe operating at a proton resonance frequency of 23.4 MHz for 16 s. Magnet temperature was maintained at 40 °C. Seed coat was removed. The samples to be measured were first dried in 105 °C oven overnight to confirm the moisture content less than 5%. The samples were weighed and maintained at 40 °C for 1 h before measurement. The oil content was measured three times by NMR against the calibration, based on the mass weight.

### Quantification of starch and soluble sugar

Starch and soluble sugar were quantified essentially as previously described [8] using 10–15-mg freeze-dried samples with four repeats, with slight modification. For sugar assay, an additional extraction was included to remove any lipid before the anthrone assay. An aliquot of 300 µL of extract was dried, dissolved in 1.2-mL chloroform:methanol (2:1), and extracted with 0.4 mL of 0.1 M KCl to recover the aqueous phase. A fraction of 20-µL extracts for caruncle or 40 µL for seed was used for soluble sugar quantification. The starch assay was done with undiluted samples.

### Microsomal preparations

Microsomal proteins were prepared from the seed and the caruncle at stage 4 as previously described [41]. All the following procedures were carried out at 0 °C. Briefly, the tissues were ground in a mortar with 5 parts (v/w) of ice-cold 0.1-M potassium phosphate buffer, pH 7.2, containing 1% bovine serum albumin (BSA), 1000 units of catalase/mL and 0.33-M sucrose. The mixture was diluted tenfold with fresh grinding medium and centrifuged at 20,000*g* for 10 min. Then, the supernatant was filtered through Miracloth^®^ and centrifuged at 105,000*g* for 90 min. The resulting microsomal pellet was re-suspended in 2 or 3 mL of potassium phosphate buffer, flash frozen in liquid nitrogen and stored at − 80 °C until use. Protein content of the microsomal preparations was measured with BCA reagents (Pierce Chemical Company) with BSA as the standard.

### Enzyme assays

TAG synthesis assay was done according to Guan et al. [42] with slight modification. Microsomal preparations (50 µg protein) were incubated at 30 °C with gentle shaking in 0.1-M Tris buffer pH 7.2 containing 4-mM MgCl_2_, 10 mg/mL fatty acid-free BSA, 12.5 nmol of [^14^C]-glycerol 3-phosphate (sp. radioactivity 8000 dpm/nmol) in a final assay volume of 50 µL. The assays were initiated by addition of 25 nmol of acyl-CoA or ricinoleoyl-CoA, in the assays with LPA substrate, and terminated by addition of 112 µL of CHCl_3_:MeOH:HAc (50:50:1) followed by extracting the lipids into a chloroform phase. All assays were performed in triplicates. Microsomal assay of fatty acid Δ12-desaturase (FAD2) and Δ12-hydroxylase (FAH12) was performed essentially as previously described [43].

### Analysis of radio-labeled lipids

The radioactivity in the chloroform phase obtained after extraction of the assay mixture was quantified by counting an aliquot by liquid scintillation. The rest of lipids were analyzed by separation on TLC plates. The plates with samples from assays with [^14^C]-glycerol 3-phosphate were developed to half height with chloroform:methanol:acetic acid:water (90:15:10:3 by vol.) to separate the polar lipids. The plates were then air dried and re-developed to the top to separate neutral lipids. In the case of TLC assays with oleoyl-CoA, the solvent mixture used for separating neutral lipids were *n*-hexane:diethyl ether:acetic acid (70:30:1 by vol.) whereas the TLC plates with assays for ricinoleoyl-CoA were re-developed with *n*-hexane:diethyl ether:acetic acid (50:50:2 by vol.). Standards of phosphatidic acid (PA), phosphatidylcholine (PC), lysophosphatidic acid (LPA), lysophosphatidylcholines (LPC), monoglyceride (MAG), diacylglycerols (DAG) and triacylglycerols (TAG) were purchased from Advanti Polar Lipids (Alabama, USA) or Sigma (Castle Hill, Australia). TAG with none, one, two and three ricinoleoyl groups were separated from castor oil by TLC with *n*-hexane:diethyl ether:acetic acid (50:50:2 by vol.) and recovered from TLC plate. Lipid standards were applied on the TLC plates to identify the different radioactive spots. The radioactive spots on the TLC plates were visualized by Phosphor Imager and their relative proportion was calculated. The absolute amount of radioactivity in each spot was calculated based on its relative amount on the plate and the total amount of ^14^C-activity in the chloroform phase.

### RNA preparation, cDNA library construction, sequencing, assembly and gene annotation

Total RNA was extracted from stage 4 developing seeds using RNeasy Mini Kit (Qiagen). RNA integrity was analyzed by capillary electrophoresis on an Agilent BioAnalyzer 2100. Library preparation and sequencing were done at John Curtin School of Medical Research, Australian National University. Equimolar concentrations of 100 bp, paired-end libraries were then sequenced separated on a single lane of the Illumine HiSeq™ 2000 system. Raw data was processed to remove adaptors, ambiguous reads, and low-quality sequences. Filtered reads were then assembled and compared with the genome sequence of *R. communis* by using CLC Genomics Workbenth 6 (http://www.clcbio.com).

### Quantitative RT-PCR

First-strand cDNA synthesis and subsequent real-time PCR were carried out in one tube using SensiFAST™ SYBR No-ROX One-Step kit (Bioline). Five nanogram/microliter template was used in this test. Reactions were carried out at one cycle of 90 °C for 2 min, 40 cycles of 95 °C for 5 s, 60 °C for 15 s, and 72 °C for 20 s. Relative normalized expression values were determined by the ΔΔC_T_ method. The expression of *RcRubisco* gene was used for normalization. All the primers are listed in Additional file 6: Table S5.

### Construct assembly and transient expression in *N. benthamiana*

Each candidate gene was recombined into Gateway binary vector pXZP393 [41]. *Agrobacterium tumefaciens* strain AGL1 was transformed with each of the constructs by electroporation. Transient expression of target genes in *N. benthamiana* leaf was performed as previously described [41]. *A. tumefaciens* cultures containing the gene coding for p19 viral suppressor protein and other gene(s) of interest were mixed. The final OD_600_ of each culture was equal to 0.3 prior to infiltration into *N. benthamiana* leaves with random triplicates. Leaf samples from infiltrated spots were harvested 5 days after infiltration and freeze dried.

### Neutral lipid quantification and fatty acid profiling by TLC and GC

Total lipids were extracted from freeze-dried *N. benthamiana* leaf samples using chloroform/methanol/KCl method [41]. TAG was fractionated by TLC (silica gel 60, Merck) in hexane:diethylether:acetic acid (70:30:1, v/v/v) and visualized by spraying Primuline (Sigma, 5 mg/100 mL 80% acetone). Silica with TAG fraction was recovered from TLC plate under UV. FAME of TAG were prepared using methanolic/HCl method [41].

## Additional files

**Additional file 1: Table S1.** Total soluble sugars and starch in developing seed and caruncle.

**Additional file 2: Table S2.** List of differentially expressed genes involved in fatty acid metabolism and TAG synthesis in the caruncle compared to in seed.

**Additional file 3: Table S3.** Gene expression level comparison by RNA-seq and qRT-PCR.

**Additional file 4: Table S4.** Fatty acid profile of leaf TAG with transit expression of the selected gene.

**Additional file 5: Figure S1.** Schematic view representing the major difference of gene expression and the distribution/channeling of the acetyl-CoA pool to fatty acid and TAG biosynthesis in caruncle and seed. The dominantly expressed gene member in the gene families identified (in bold) are highlighted in blue for caruncle and dark red for seed. In the case of oleosin genes, oleosin 1 was the dominant gene in both caruncle and seed, but lower in caruncle (indicated by non-bold) compared to seed. The genes for other steps that no significant difference identified here are not shown. The major fatty acids in the pathway and the ultimate profile in TAG are also indicated in blue for caruncle and dark red for seed. RA, ricinoleic acid (12OH-C18:1^∆9^). Other abbreviations are same as in Fig. 5.

**Additional file 6: Table S5.** Primers used for qRT-PCR.

## Data Availability

All data generated or analyzed during this study are included in this article and its additional files.

## Abbreviations

ACCase: acetyl-CoA carboxylase
ACP: acyl carrier protein
ACS: acyl-CoA synthetase
BSA: bovine serum albumin
CoA: coenzyme A
CPT: cholinephosphotransferase
DAG: diacylglycerol
DGAT: acyl-CoA:diacylglycerol acyltransferase
DGDG: digalactosyldiacylglycerol
FAD2: fatty acid desaturase 2 (Δ12-desaturase)
FAD3: fatty acid desaturase 3 (Δ15-desaturase)
FAH12: Δ12-hydroxylase
FAME: fatty acid methyl esters
FATA or FATB: acyl acyl-carrier-protein thioesterase A or B
FFA: free fatty acid
FPKM: fragments per kilobase per million mapped reads
G3P: glycerol-3-phosphate
GC: gas chromatography
GPAT: glycerol-3-phosphate acyltransferase
LACS: long-chain acyl-CoA synthetase
LDAP: lipid droplet-associated protein
LPA: lysophosphatidic acid
LPAAT: lysophosphatidic acid acyltransferase
LPC: lysophosphatidylcholine
LPCAT: lysophosphatidylcholine acyltransferase
MAG: monoacylglycerol
MGDG: monogalactosyldiacylglycerol
NMR: nuclear magnetic resonance
PA: phosphatidic acid
PAP: phosphatidic acid phosphatase
PC: phosphatidylcholine
PDAT: phospholipid:diacylglycerol acyltransferase
PDCT: phosphatidylcholine:diacylglycerol cholinephosphotransferase
PLA2: phospholipase A2
PLC: phospholipase C
PLD: phospholipase D
RA: ricinoleic acid
SAD: stearoyl-ACP desaturase
TAG: triacylglycerol
TLC: thin layer chromatography
WRI: WRINKLED transcription factor
