# Supplementary figures and images for: Molecular and biochemical analysis of the castor caruncle reveals a set of unique genes involved in oil accumulation in non-seed tissues
Source: Biotechnol Biofuels. 2019 Jun 24;12:158. doi: 10.1186/s13068-019-1496-6 (PMC6589891; doi:10.1186/s13068-019-1496-6)

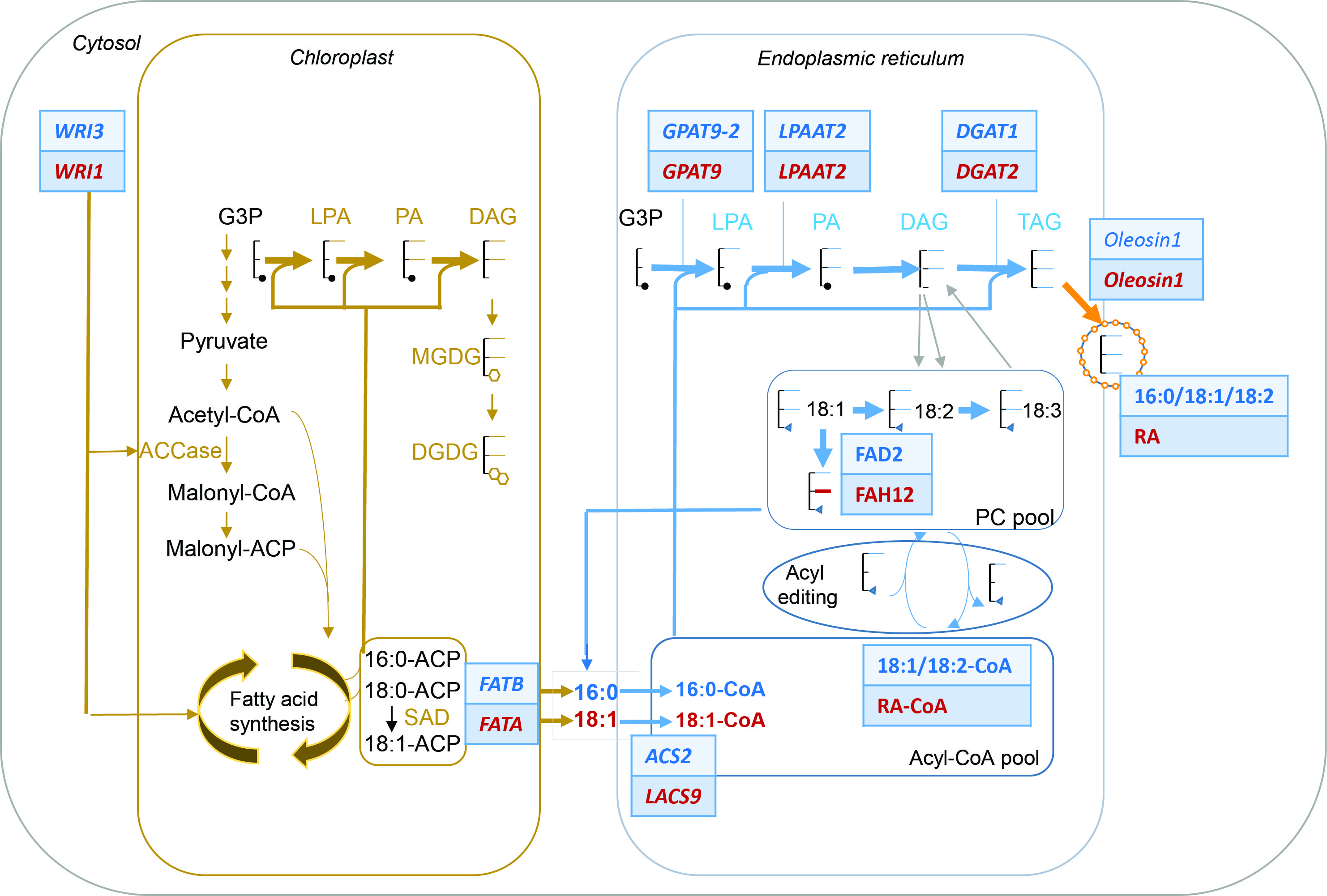

Supplement: Supplementary file 5 — Additional file 5: Figure S1. Schematic view representing the major difference of gene expression and the distribution/channeling of the acetyl-CoA pool to fatty acid and TAG biosynthesis in caruncle and seed. The dominantly expressed gene member in the gene families identified (in bold) are highlighted in blue for caruncle and dark red for seed. In the case of oleosin genes, oleosin 1 was the dominant gene in both caruncle and seed, but lower in caruncle (indicated by non-bold) compared to seed. The genes for other steps that no significant difference identified here are not shown. The major fatty acids in the pathway and the ultimate profile in TAG are also indicated in blue for caruncle and dark red for seed. RA, ricinoleic acid (12OH-C18:1∆9). Other abbreviations are same as in Fig. 5. [file 13068_2019_1496_MOESM5_ESM.jpg]
